# A dose escalating phase I study of GLPG0187, a broad spectrum integrin receptor antagonist, in adult patients with progressive high-grade glioma and other advanced solid malignancies

**DOI:** 10.1007/s10637-015-0320-9

**Published:** 2016-01-20

**Authors:** Geert A. Cirkel, Bojana Milojkovic Kerklaan, Frédéric Vanhoutte, Annegret Van der Aa, Giocondo Lorenzon, Florence Namour, Philippe Pujuguet, Sophie Darquenne, Filip Y. F. de Vos, Tom J. Snijders, Emile E. Voest, Jan H. M. Schellens, Martijn P. Lolkema

**Affiliations:** Department of Medical Oncology, University Medical Center Utrecht, Heidelberglaan 100, 3584 CX Utrecht, The Netherlands; Department of Clinical Pharmacology, Netherlands Cancer Institute, Plesmanlaan 121, 1066 CX Amsterdam, The Netherlands; Galapagos NV, Gen. De Wittelaan L11A3, 2800 Mechelen, Belgium; Galapagos SASU, 102 Avenue Gaston Roussel, 93230 Romainville, France; Brain Center Rudolf Magnus, Department of Neurology and Neurosurgery, University Medical Center Utrecht, Heidelberglaan 100, 3584 CX Utrecht, The Netherlands; Erasmus MC Kanker Instituut, Groene Hillededijk 301, Room G4-51, 3075 EA Rotterdam, The Netherlands; Netherlands Cancer Institute, Plesmanlaan 121, PO box: 90203, 1006 BE Amsterdam, The Netherlands

**Keywords:** Integrin, Antagonist, Glioma, Phase 1, GLPG0187

## Abstract

*Background* Integrin signaling is an attractive target for anti-cancer treatment. GLPG0187 is a broad spectrum integrin receptor antagonist (IRA). GLPG0187 inhibited tumor growth and metastasis in mouse models. *Methods* We aimed to determine the Recommended Phase II Dose (RP2D) and to assess safety and tolerability of continuous i.v. infusion in patients with advanced malignant solid tumors. Anticipated dose levels were 20, 40, 80, 160, 320, and 400 mg/day in a modified 3 + 3 design. Plasma concentrations of GLPG0187 were assessed to characterize the pharmacokinetics (PK). C-terminal telopeptide of type I collagen (CTX) was used as pharmacodynamics marker. *Results* Twenty patients received GLPG0187. No dose limiting toxicities (DLTs) were observed. The highest possible and tested dose was 400 mg/day. Fatigue was the most frequently reported side effect (25 %). Recurrent Port-A-Cath-related infections and skin toxicity suggest cutaneous integrin inhibition. No dose-dependent toxicity could be established. PK analysis showed a short average distribution (0.16 h) and elimination (3.8 h) half-life. Continuous infusion resulted in dose proportional PK profiles. We observed decreases in serum CTX levels independent of the dose given, suggesting target engagement at the lowest dose level tested. Single agent treatment did not result in tumor responses. *Conclusions* GLPG0187 was well tolerated with a dose-proportional PK profile upon continuous infusion. No formal maximal tolerated dose could be established. GLPG0187 showed signs of target engagement with a favourable toxicity profile. However, continuous infusion of GLPG0187 failed to show signs of monotherapy efficacy.

**Electronic supplementary material:**

The online version of this article (doi:10.1007/s10637-015-0320-9) contains supplementary material, which is available to authorized users.

## Introduction

Integrin signaling plays an important role in cancer biology providing a strong rationale to pursue integrin receptor antagonists (IRA) as therapeutic agents in cancer patients [1, 2]. Integrin receptors are heterodimeric cell surface molecules that act as adhesion molecules connecting the cytoskeleton to the extracellular matrix. Moreover they are involved in activating intracellular signaling pathways, actin cytoskeleton remodelling, three-dimensional cell growth and metastatatic organotropism [3, 4]. Integrin-mediated signaling is implicated in modulation of well-known cancer-related pathways such as the TGF-beta pathway in glioblastoma and the Rho-Rac pathway [2, 5, 6].

Cilengitide is the most advanced IRA in clinical development. Cilengitide showed signs of efficacy without significant additive toxicity both as single agent and combined with radiation and temozolomide in patients with glioblastoma multiforme (GBM) [7–9]. Unfortunately cilengitide failed to live up to its promise in a phase III clinical trial when combined with standard treatment in GBM and further clinical development seems unlikely [10–12]. When compared to cilengitide, GLPG0187 has a stronger nanomolar affinity for a broader panel of RGD (Arg-Gly-Asp) integrin receptors (αvβ1, αvβ3, αvβ5, αvβ6, αvβ8 and α5β1; supplementary table S1 and [13, 14]). In preclinical models GLPG0187 significantly inhibited angiogenesis both in vivo and in vitro, osteoclastogenesis in vitro, and bone loss in vivo [15]. In mouse cancer models GLPG0187 inhibited de novo formation and progression of bone and visceral metastases in prostate cancer and breast cancer [13, 14, 16, 17]. We hypothesized that GLPG0187, a more potent and broader spectrum IRA when compared to cilengitide, may improve the anti-tumor efficacy of IRA therapy. Therefore a phase I dose escalation study was initiated to investigate the safety and tolerability of GLPG0187 when administered intravenously in end-stage cancer patients. In healthy volunteers, GLPG0187 was rapidly eliminated after oral administration with a terminal half-life of about 5–6 h [18]. To ensure continuous target inhibition despite its relatively short half-life GLPG0187 was administered as a continuous i.v. infusion in this study. We aimed to determine a safe dose in cancer patients and to determine the pharmacokinetics (PK), pharmacodynamics (PD) and evaluate preliminary signs of efficacy.

## Materials and methods

### Patient selection

Patients, aged over 18 years, with pathologically confirmed advanced or metastatic malignant solid tumors refractory to standard therapy or for whom no standard treatment options were available were eligible for participation. Additional inclusion criteria included: written informed consent, measurable disease according the Response Evaluation Criteria In Solid Tumors (RECIST) version 1.1 [19], Eastern Cooperative Oncology Group (ECOG) performance status of 0–2, estimated life expectancy of at least 12 weeks and no previously incurred anticancer therapy related toxicities higher than grade 2. Patients were considered ineligible if there was less than 4 weeks since their last anticancer therapy (less than 6 weeks for nitrosoureas and mitomycin C) or they were previously treated with IRAs. Additional exclusion criteria were: chronic treatment with corticosteroids equivalent to 10 mg methylprednisolone per day or more, current or recent (within 30 days) treatment with another investigational drug or participation in another investigational study, clinically symptomatic or progressive brain or leptomeningeal metastases, major surgical procedure within 28 days before first dose, congestive heart failure (NYHA class 3 or 4), clinical significant cardiac arrhythmias, signs and symptoms of relevant cardiovascular disease, known hypersensitivity to any of the study drugs and significant medical conditions possibly interfering with patient compliance or safe study participation.

Female patients with reproductive potential were only eligible with a negative pregnancy test obtained less than 7 days before first dose and if an adequate contraceptive method was used while on study. There were no restrictions in concomitant medication.

The study was centrally approved by the ethics committee of the University Medical Center Utrecht and was conducted according to the Declaration of Helsinki and Good Clinical Practice guidelines. Written informed consent was obtained from all patients. The study was registered on clinicaltrials.gov (NCT01313598).

### Investigational agent

GLPG0187 supply was controlled by Galapagos SASU (Romainville, France) and was delivered to participating sites as a 35 mg/ml injectable solution in type 1 clear glass vials. A 40 % HP-β-CD (Kleptose®) injectable solution manufactured by Roquette pharma (Lestrem, France) was used to improve solubility of GLPG0187. Depending on dose level, various amounts of GLPG0187 were dissolved in HP-β-CD injectable solution and saline and administered by continuous infusion to patients at the recommended infusion rate. The amount of HP-β-CD needed to prepare a 400 mg/day GLPG0187 solution (around 8 g/day) was considered the maximum acceptable dose in humans based on the clinical experience with itraconazole solved in HP-β-CD. No dose escalation beyond 400 mg/day was planned within this study. After preparation, the solution was stored at room temperature protected from daylight for a maximum of 7 days.

### Study design

This study was performed as a multicenter, open-label, dose-escalation, phase I study. Patients were accrued in the University Medical Center Utrecht and The Netherlands Cancer Institute. Decisions regarding dose escalation were made by using a modified 3 + 3 dose escalation design. To reduce the number of patients treated at possible suboptimal dose concentrations, only 2 evaluable patients were assigned in the first two cohorts.

A small wearable infusion pump (Pega plus infusion pump, Venner Medical, Ecublens,

Switzerland) enabled continuous GLPG0187 infusion at home through a Port-A-Cath (PAC) or peripherally inserted central catheter (PICC). Renewals of the infusion pumps and bags were performed at weekly hospital visits with at the higher dose levels additional renewals at home by specialized nurses. With these measures continuous infusion was possible with as little as possible impact on participants daily lives and wellbeing.

Patients in the first cohort received a starting dose of 20 mg/day which was chosen based on results from a preceding healthy volunteer study [18]. The anticipated sequential dose escalations were 20, 40, 80, 160, 320 and 400 mg/day. Intrapatient dose-escalations were not allowed. Dose reductions or interruptions were allowed after cycle 1 which equals the dose limiting toxicity (DLT) window.

On day 1 of cycle 1, a single daily dose of GLPG0187 was administered at a constant infusion rate for a period of 1 hour after which the patient was followed for 24-h to assess the PK profile. On day 8 of cycle 1 continuous infusion was initiated at the assigned dose level for 21 days. Thereafter, treatment was continued uninterrupted in 21-day cycles until disease progression, occurrence of a DLT, unacceptable toxicity, death, poor study compliance or withdrawal of informed consent.

A DLT was defined as one of the following adverse events (AEs) considered related to GLPG0187 occurring within the first cycle of 28 days: grade 4 neutropenia lasting ≥7 consecutive days, febrile neutropenia (defined as absolute neutrophil count (ANC) ≤ 1000 cells per μL and fever ≥38.5 °C) or documented infection ≥ grade 3 with ANC ≤ 1000 cells/μL, grade 4 thrombocytopenia, thrombocytopenia requiring platelet transfusion, or bleeding requiring medical intervention, alanine aminotransferase (ALT) or aspartate aminotransferase (AST) > 5 × upper limit of normal (ULN) (> 7.5 × ULN in case of liver metastases) for greater than 14 days, ALT or AST > 5 × ULN (> 7.5 × ULN in case of liver metastases) co-occurring with a total bilirubin of >2.5 × ULN (not explained by obstruction) regardless of duration, non-hematological toxicity ≥ grade 3. GLPG0187-related nausea, vomiting, and diarrhoea were only considered DLTs if they persisted at ≥ grade 3 for >3 days despite adequate supportive care measures.

The Maximum Tolerated Dose (MTD) for this study was defined as the dose level below the dose level at which ≥2 patients in a dose cohort experienced a DLT within the DLT observation period. The resulting recommended phase II dose (RP2D) was defined as the MTD or the highest tested dose which is tolerable and safe.

### Safety and efficacy assessments

After signing informed consent, patients were screened for eligibility. Screening assessments were performed within 14 days of the first dose.

Safety was assessed weekly by means of physical examination, weight, vital signs, ECOG performance status, laboratory evaluations (hematology, biochemistry and urinalysis), electrocardiograms, and recording of concurrent illness/therapy and AEs throughout the study course. An AE was defined as appearance of any (or worsening of any preexisting) undesirable sign, symptom or medical condition occurring after signing the informed consent, whether related to treatment or not. Toxicity was graded according to the National Cancer Institute Common Terminology Criteria for Adverse Events (NCI-CTCAE) version 4.03. For each AE an absent, unlikely, possible, probable or certain relationship with GLPG0187 was assessed by the local investigator.

Preliminary efficacy was measured bi-cyclic and at end of treatment by CT scan, MRI, or a bone scan following RECIST 1.1 [19]. Recent literature has highlighted the need for better criteria for response assessment in high-grade gliomas, and the Response Assessment in Neuro-oncology (RANO) group has published new MRI-based response criteria [20]. For this reason, we evaluated all high-grade glioma patients according to RANO criteria. Concordance between RANO and RECIST evaluation of all gliomas was 100 %.

### Pharmacokinetic and pharmacodynamic methods

PK blood samples were collected at baseline and on day 1 of cycle 1 at 0.5, 1, 1.5, 2, 4, 6, 8 and 24 h after start of the single first dose. Additional samples were obtained on day 8, 15, 22 and 28 of cycle 1. PK samples were analyzed for determination of GLPG0187 plasma levels by a validated liquid chromatography-mass spectrometry methods at AtlanBio (Saint-Nazaire, France). PK parameters in plasma such as maximum concentration (C_max_), Area Under the Curve (AUC), total plasma clearance, steady state volume of distribution (V_ss_) and distribution and elimination half-lives (t_1/2lbd1_ and t_1/2lbdz_) were calculated, as well as dose standardized parameters (C_max_/dose and AUC/dose).

Integrin signaling is crucial for bone resorption by osteoclasts [21, 22]. In a study performed by van der Horst et al. GLPG0187 inhibited osteoclastic bone resorption in mice significantly [15]. In addition, GLPG0187 was shown to significantly reduce CTX (C-terminal telopeptide of type I collagen) levels when compared with placebo in a phase I healthy volunteer study [18]. Therefore CTX, a well-established marker for bone turn-over, was adopted as surrogate PD marker in this study [23]. CTX serum levels were measured by ELISA, according to the manufacturer instructions (CrossLaps, Immuno Diagnostic Systems, ref. AC-02F1).

CTX levels were measured in blood samples collected at baseline and on day 1 of cycle 1 at 1, 2, 4, 6, 8 and 24 h after start of the first single dose. Additional samples were obtained on day 8, 15, 22 and 28 of cycle 1. These samples were obtained in the morning in a fasting state.

### Statistical methods

Study results were obtained by analyzing the safety population which contains all patients who received at least 1 dose of GLPG0187. Results were summarized descriptively and if applicable plotted by dose level over time. CTX levels at different time points were compared by using a Wilcoxon Signed Rank Test.

## Results

### Patients

Twenty patients received GLPG0187, between 22nd of March 2011 and 10th of April 2013. Patient characteristics are depicted in Table 1. Fifteen patients completed cycle 1 and were considered evaluable for DLT assessment. Patients with progressive high-grade glioma were most commonly included (40 %, GBMs) based on pre-clinical and early phase clinical data available [24, 7, 25].

**Table 1:** Patient demographics

	Total (*N* = 20) N (%)
Age (years)	
Mean (SD) Median (range)	56.4 (11.9) 58,5 (35–76)
Gender	
Male	14 (70.0)
Female	6 (30.0)
ECOG	
0	4 (20.0)
1	11 (55.0)
2	5 (25.0)
Ethnicity	
Caucasian/white	19 (95.0)
Black	1 (5.0)
Primary tumor	
High-grade gliomas	
Glioblastoma multiforme	5 (25)
Anaplastic oligodendroglioma	2 (10)
High grade astrocytoma^a^	1 (5)
Colorectal carcinoma	3 (15)
Adenocarcinoma of Unknown Primary	1 (5)
Adenoid cystic carcinoma	1 (5)
Cholangiocarcinoma	1 (5)
Endometrial cancer	1 (5)
Nasopharynxcarcinoma	1 (5)
Non-small cell lung cancer	1 (5)
Ocular Melanoma	1 (5)
Osteosarcoma	1 (5)
Urothelial cell carcinoma	1 (5)

*N* number of patients, *ECOG* Eastern Cooperative Oncology Group performance status

^a^Secondary form, from low grade astrocytoma

### Dose escalation and safety

No DLTs were observed in any cohort. The absence of DLTs resulted in an undisturbed dose escalation scheme towards the final planned cohort of 400 mg/day. No MTD could be established.

GLPG0187 showed a tolerable toxicity profile in this study. The incidence of at least possibly related AEs per cohort is summarized in Table 2. Most frequently observed toxicities were fatigue (5 patients, 25 %) and skin related adverse events (5 patients, 25 %). Twenty-three AEs were considered possibly related and 6 probably related to GLPG0187. All but two AEs are reported only once. During the study, 14 (70 %) patients experienced a total of 23 serious adverse events (SAE). Only one SAE was considered possibly related (fatigue). All other SAEs were assessed as unlikely or not-related.

**Table 2:** All and ≥ Grade 3 at least possibly GLPG0187-related AEs per dose cohort

	20 mg/day		40 mg/day		80 mg/day		160 mg/day		320 mg/day		400 mg/day		Total	
	(*N* = 2)		(*N* = 5)		(*N* = 4)		(*N* = 3)		(*N* = 3)		(*N* = 3)		(*N* = 20)	
Adverse event description^a^	All	≥Gr 3	All	≥Gr 3	All	≥Gr 3	All	≥Gr 3	All	≥Gr 3	All	≥Gr 3	All	≥Gr 3
Dry mouth	1	0	0	0	0	0	0	0	0	0	0	0	1	0
Epidermolysis	0	0	0	0	1	0	0	0	0	0	0	0	1	0
Fungal skin infection	0	0	0	0	0	0	0	0	1	0	0	0	1	0
Herpes zoster	0	0	0	0	1	0	0	0	0	0	0	0	1	0
Mucosal inflammation	1	0	0	0	0	0	0	0	0	0	0	0	1	0
Rash	0	0	1	0	0	0	0	0	0	0	0	0	1	0
Rash Maculo-papular	1	0	0	0	0	0	0	0	0	0	0	0	1	0
Skin hyperpigmentation	1	0	0	0	0	0	0	0	0	0	0	0	1	0
Arthralgia	1	0	0	0	0	0	0	0	0	0	0	0	1	0
Conjunctival Haemorrhage	0	0	0	0	0	0	0	0	0	0	1	0	1	0
Diarrhoea	1	0	0	0	0	0	0	0	0	0	0	0	1	0
Dysgeusia	0	0	0	0	0	0	0	0	0	0	1	0	1	0
Fatigue	0	0	0	0	2	0	1	0	1	1	1	0	5	1
Headache	0	0	0	0	1	0	0	0	0	0	0	0	1	0
Oedema peripheral	0	0	0	0	0	0	0	0	1	0	0	0	1	0
Peripheral sensory neuropathy	1	0	0	0	0	0	0	0	0	0	0	0	1	0
Pleural effusion	0	0	0	0	0	0	0	0	1	0	0	0	1	0
Thrombosis	0	0	0	0	1	0	0	0	0	0	0	0	1	0
Vasculitis	0	0	1	0	0	0	0	0	0	0	0	0	1	0
ALT increased	0	0	0	0	1	1	0	0	0	0	0	0	1	1
Anemia	0	0	1	0	0	0	0	0	0	0	0	0	1	0
Blood albumin decreased	0	0	2	0	0	0	0	0	0	0	0	0	2	0
Blood bilirubin increased	0	0	1	0	0	0	0	0	0	0	0	0	1	0
Blood creatinine increased	0	0	1	0	0	0	0	0	0	0	0	0	1	0
Total	7	0	7	0	7	1	1	0	4	1	3	0	29	2

*ALT* Alanine aminotransferase, *N* number of patients

^a^Adverse events were evaluated using the National Cancer Institute Common Toxicity Criteria for Adverse Events, version 4.03

All toxicity seemed manageable and did not lead to dose reductions or dose interruptions. No clear relationship was observed between GLPG0187 dose level and the occurrence of AEs or laboratory abnormalities.

### Pharmacokinetic data

**Fig. 1:**
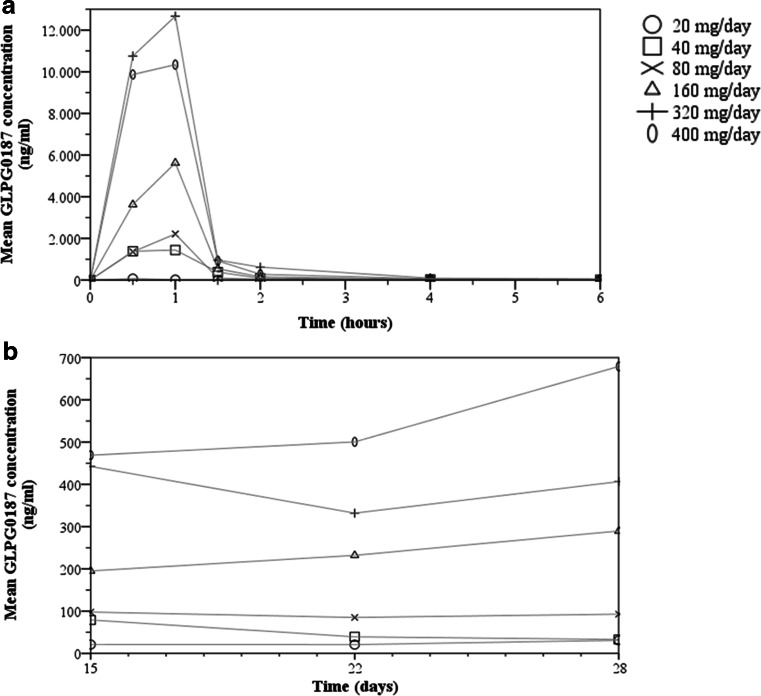
**a** The graph depicts the mean plasma concentration of GLPG0187 over the first 6 h after GLPG0187 treatment start. GLPG0187 concentration observed at 8 and 24 h post-infusion was below the limit of quantification. **b** The graph depicts the mean plasma concentration of GLPG0187 over 14 days during continuous GLPG0187 i.v. infusion. Plasma concentrations on day 8 were below the level of quantification

After intravenous infusion, GLPG0187 was rapidly distributed and eliminated as illustrated in Fig. 1a. The PK profile was dose proportional over the 20 to 400 mg/day dose range when infused continuously (Fig. 1b). PK parameters per cohort are displayed in Table 3. GLPG0187 showed a moderate total plasma clearance (average: 40.1 L/h) and short distribution and elimination half-lives of on average 0.16 and 3.8 h, respectively. GLPG0187 plasma concentration was maintained during the PK sampling period of 21 days while receiving continuous i.v. infusion (Fig. 1b).

**Table 3:** Mean PK (SD) parameters per dose cohort

Parameter	20 mg/day (*N* = 2)	40 mg/day (*N* = 5)	80 mg/day (*N* = 4)	160 mg/day (*N* = 3)	320 mg/day (*N* = 3)	400 mg/day (*N* = 3)
AUC _(0-inf)_ (ng.h/mL)	422	1689 (822)	1866 (387)	4357 (1764)	12,386 (3951)	10,114 (6693)
AUC _(0-inf)/dose_ (ng.h/mL)	21.1	47.3 (18.4)	23.3 (4.8)	27.2 (11.0)	38.7 (12.3)	25.3 (16.7)
t_1/2,lbd1_ (h)	0.182	0.180 (0.097)	0.117 (0.045)	0.165 (0.060)	0.148 (0.022)	0.140 (0.030)
t_1/2,lbdz_ (h)	2.41	2.98 (1.01)	3.92 (1.27)	4.68 (0.35)	5.07 (0.26)	3.44 (1.11)
C_max_ (ng/mL)	391	1460 (677)	1751 (396)	3969 (1511)	11,490 (3821)	9592 (6340)
C_max/dose_ (ng/mL.mg)	19.5	41.1 (15.5)	21.9 (5.0)	24.8 (9.4)	35.9 (11.9)	24.0 (15.9)
CL (L/h)	48.7	24.3 (10.3)	44.3 (9.2)	42.5 (21.6)	27.6 (8.2)	53.2 (33.3)
Vss (L)	25.6	15.4 (13.0)	25.7 (17.3)	26.1 (2.2)	20.6 (7.1)	23.0 (14.5)

*N* number of patients, *AUC* area under the curve, t_1/2,lbd1_ distribution half- life, t_1/2,lbdz_ terminal elimination half- life, C_max_ maximum concentration, *CL* clearance, *Vss* steady state volume of distribution

### Effects on bone resorption marker CTX

**Fig. 2:**
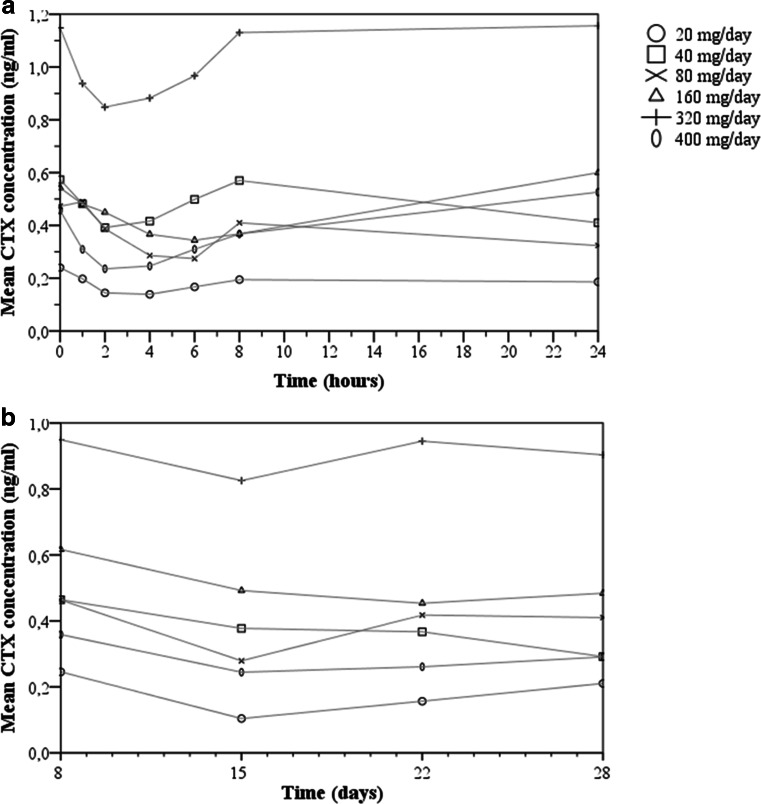
**a** Mean concentration of bone resorption marker CTX over the first 24 h after a single i.v. dose of GLPG0187. **b** Mean CTX concentration measured on day 8, 15, 22 and 28. Continuous infusion of GLPG0187 was initiated on day 8

The effect of GLPG0187 treatment on CTX levels was measured in serum during the first cycle and is depicted in Fig. 2a/b. High intra- and interpatient variability in the CTX concentration measurements was observed. The presence of bone metastases in 3 patients was not explanatory for the variability observed. A Wilcoxon Signed Rank Test was conducted to compare CTX levels of the total study population at baseline to 2 h post infusion on cycle 1 day 1. Additionally, the effect of continuous infusion was analyzed by comparing mean CTX levels at day 15 to day 8. A significant change in CTX level was observed 2 h after the single dose infusion on day 1. The mean CTX level of the total study population was higher at baseline: 0.58 ng/ml (SD 0.39) versus 0.42 ng/ml (SD 0.32), *p* < 0.0001. At day 15 CTX levels were lower compared to day 8 (*p* = 0.007). No relationship between GLPG0187 dose and change in CTX concentration was observed within cycle 1.

### Efficacy

Stable disease was observed in 3 (15 %) out of 20 patients. These 3 patients were treated at 20 mg/day (patient with non-small cell lung cancer, stable disease during 14 weeks), 80 mg/day (patient with GBM, stable disease during 19 weeks) and 160 mg/day (patient with GBM, stable disease during 8 weeks). No tumor responses were observed.

## Discussion

We performed a phase I, open-label, dose escalation study using GLPG0187 in patients with solid tumors. GLPG0187 was well-tolerated and displayed a predictable and dose proportional PK profile. The toxicity profile of GLPG0187 when given as a continuous iv infusion is mild at the maximal administered dose and we did not identify a maximal tolerated dose.

Although no directly related severe toxicity was observed we did observe PAC related infections in three out of six patients in the first two cohorts. They received GLPG0187 through a PAC system. As the median reported infection rate of totally implantable intravenous catheter devices within an immunosuppressed population approximates 0.2 per 1000 catheter days [26] we consider these events retrospectively as possibly related to GLPG0187. For all three patients the infection was evident at the skin location where the needle enters the skin. From cohort 3 onwards we switched to a PICC, where access to the system does not require repeated skin punctures. No catheter related infections were observed after the switch from PAC to PICC. These observations combined with the fact that skin toxicity was the most common side effect found suggest that skin integrity may be compromised in the presence of GLPG0187. The observed changes in CTX levels indirectly suggest that GLPG0187 does inhibit integrin function. Despite the baseline variation we found a statistically significant decrease in CTX levels after GLPG0187 administration. Bone turnover, and associated markers including CTX, follow a circadian rhythm with peak levels at night and a decrease towards nadir in the afternoon [27, 28]. All patients received their single dose on day 1 in the morning. Therefore, on day 1 we cannot exclude the possibility that CTX levels in our patients partially, if not completely, decreased by a physiological phenomenon rather than a PD effect. However, the clear decrease at day 15 cannot be explained in this manner and most likely represents a treatment effect. Thus, both toxicity and PD correlative markers suggest a treatment dependent inhibition of physiological integrin signaling.

During this study the development of cilengitide, the first generation IRA stopped due to a negative trial in patients with high-grade glioma [10, 11]. These disappointing results for cilengitide may reflect either inherent inefficacy of cilengitide or problems with dose, scheduling and patient selection. Regarding dose and scheduling it appears that more could be less and intermittent scheduling could have more efficacy than more intensive schedules [12]. Here we explored the extreme of exposure driven therapy: continuous iv infusion. Although continuous exposure seems to be important in treatment targeting driving oncogenic pathways such as BRAF or EGFR it is questionable whether integrin signaling could be classified as such. Authors have proposed that inadequate exposure may in part explain the lack of efficacy of cilengitide [29]. Our data suggest that even continuous iv infusion of a more potent IRA does not result in the expected efficacy signals.

The development of IRAs in oncology has been largely disappointing and our study suggests that the lack of efficacy cannot easily be attributed to exposure, completeness of integrin signaling blockade or affinity issues of the first generation compounds. Indeed the pre-clinical models have failed to lead the way for rational drug design and rational trial design for IRAs. The data known for cilengitide in the clinical setting warrants a discontinuation of IRA development until novel more relevant combination strategies have been designed that can be tested in small proof-of-principle clinical trials.

## Electronic supplementary material

Supplementary Table S1 (DOCX 14 kb)
