# Supplementary material for: A dose escalating phase I study of GLPG0187, a broad spectrum integrin receptor antagonist, in adult patients with progressive high-grade glioma and other advanced solid malignancies
Source: Invest New Drugs. 2016 Jan 20;34:184–92. doi: 10.1007/s10637-015-0320-9 (PMC4786599; doi:10.1007/s10637-015-0320-9)
Supplement: Supplementary file 1 — (DOCX 14 kb) [file 10637_2015_320_MOESM1_ESM.docx]

# A dose escalating phase I study of GLPG0187, a broad spectrum integrin receptor antagonist, in adult patients with progressive high-grade glioma and other advanced solid malignancies.

*Investigational new drugs*

Geert A. Cirkel^1^, Bojana Milojkovic Kerklaan^2^, Frédéric Vanhoutte^3^, Annegret Van der Aa^3^, Giocondo Lorenzon^4^, Florence Namour^4^, Philippe Pujuguet^4^, Sophie Darquenne^4^, Filip Y.F. de Vos^1^, Tom J. Snijders^5^, Emile E. Voest^1,6^, Jan H.M. Schellens^2^, Martijn P. Lolkema^1,7^

^1^Department of Medical Oncology, University Medical Center Utrecht, The Netherlands

^2^Department of Clinical Pharmacology, Netherlands Cancer Institute, The Netherlands

^3^Galapagos NV, Belgium

^4^Galapagos SASU, France

^5^Brain Center Rudolf Magnus, department of Neurology and Neurosurgery, University Medical Center Utrecht, The Netherlands

^6^Department of Medical Oncology, Netherlands Cancer Institute, The Netherlands

^7^Corresponding author: email: [m.lolkema@erasmusmc.nl](mailto:m.lolkema@erasmusmc.nl)

### Table S1: Comparative affinities for different integrin receptors^a^ (IC_50_, as nanomoles/L)

|  | **αvβ1** | **αvβ3** | **αvβ5** | **αvβ6** | **avβ8** | **α5β1** |
| --- | --- | --- | --- | --- | --- | --- |
| **Cilengitide** | 11.2 ± 3.7 | 6.5 ± 2.3 | 5.2 ± 1.3 | 122 ± 14 | 436 ± 85 | 30.5 ± 3.3 |
| **GLPG0187** | 1.3 ± 0.4 | 3.7 ± 0.6 | 2.0 ± 0.6 | 1.4 ± 0.3 | 1.2 ± 0.3 | 7.7 ± 4.0 |
| ^a^: Competitive binding (in a solid phase assay) | | | | | | |
